# Computational Modelling of NF-κB Activation by IL-1RI and Its Co-Receptor TILRR, Predicts a Role for Cytoskeletal Sequestration of IκBα in Inflammatory Signalling

**DOI:** 10.1371/journal.pone.0129888

**Published:** 2015-06-25

**Authors:** David M. Rhodes, Sarah A. Smith, Mike Holcombe, Eva E. Qwarnstrom

**Affiliations:** 1 Department of Cardiovascular Science, Medical School, University of Sheffield, United Kingdom; 2 Department of Computer Science, University of Sheffield, Sheffield, United Kingdom; IISER-TVM, INDIA

## Abstract

The transcription factor NF-κB (nuclear factor kappa B) is activated by Toll-like receptors and controlled by mechanotransduction and changes in the cytoskeleton. In this study we combine 3-D predictive protein modelling and *in vitro* experiments with *in silico* simulations to determine the role of the cytoskeleton in regulation of NF-κB. Simulations used a comprehensive agent-based model of the NF-κB pathway, which includes the type 1 IL-1 receptor (IL-1R1) complex and signalling intermediates, as well as cytoskeletal components. Agent based modelling relies on *in silico* reproductions of systems through the interactions of its components, and provides a reliable tool in investigations of biological processes, which require spatial considerations and involve complex formation and translocation of regulatory components. We show that our model faithfully reproduces the multiple steps comprising the NF-κB pathway, and provides a framework from which we can explore novel aspects of the system. The analysis, using 3-D predictive protein modelling and *in vitro* assays, demonstrated that the NF-κB inhibitor, IκBα is sequestered to the actin/spectrin complex within the cytoskeleton of the resting cell, and released during IL-1 stimulation, through a process controlled by the IL-1RI co-receptor TILRR (Toll-like and IL-1 receptor regulator). *In silico* simulations using the agent-based model predict that the cytoskeletal pool of IκBα is released to adjust signal amplification in relation to input levels. The results suggest that the process provides a mechanism for signal calibration and enables efficient, activation-sensitive regulation of NF-κB and inflammatory responses.

## Introduction

The transcription factor NF-κB is central to control of inflammatory responses and anti-apoptotic signals. Dys-regulation of the system underlies chronic inflammatory diseases and tumour development [1–3]. In the resting cell, NF-κB is sequestered in the cytoplasm by inhibitors of NF-κB [4]. Activation of NF-κB through the canonical pathway involves rapid phosphorylation, ubiquitination and degradation of the key inhibitor, IκBα allowing NF-κB to enter the nucleus and trigger gene transcription. NF-κB induction of IκBα provides a negative feedback, which is crucial to pathway control. Activation of NF-κB is induced by members of the Toll-like and IL-1 receptor (TIR) family and regulated in part through changes in cytoskeletal components and mechanotransduction [5–10].

Our earlier studies have demonstrated that under resting conditions about 2/3 of the NF-κB inhibitor, IκBα, are sequestered by the cytoskeleton [11]. Of interest in this study is the role of cytoskeletal bound IκBα in activation of NF-κB by the cytokine IL-1. Specifically, we assess the interaction of the inhibitor with the cytoskeletal component spectrin [12]. Three-D protein modelling and docking interactions, together with *in vitro* experiments were used to predict the relationship between IκBα and the cytoskeletal components, and to monitor its dissociation from the complex during pathway activation. Subsequent analysis used a comprehensive agent based model, including the IL-1 receptor complex, signalling intermediates and cytoskeletal components to assess the impact of the cytoskeletal inhibitor pool on NF-κB activation.

Our data show binding of IκBα to the cytoskeleton, demonstrate release of the inhibitor from the cytoskeletal complex during NF-κB activation in a process controlled by the IL-1RI co-receptor TILRR [13,14], and predict a distinct role for this mechanism in regulation of receptor activated signal amplification.

## Materials and Methods

### Computational modelling

#### Agent Based Simulations

Agent based modelling is a highly detailed and flexible tool, which enables *in silico* reproductions of systems with large numbers of similar entities, such as regulatory proteins in a signalling pathway (S2–S5 Texts, S1–S3 Tables). Agents are autonomous entities with specific states and behaviour, governed by rules, which determine their interactions with the environment and with other agents in the system. *In silico* experiments use an agent-based model, which is an expansion of our earlier model, and which we show faithfully describes the biological system [11]. The agent based model utilises a three dimensional space in which each agent has a specific location at any given time and can only interact with other agents within its local vicinity. Hence each adaptor protein must move to the location of an activated receptor in order to itself become activated and initiate the signalling cascade. Similarly, proteins such as transcription factors must move to the location of a nuclear import or export receptor in order to translocate between cytoplasm and nucleus. Once in the nucleus it needs to move into interaction range with a transcription site to trigger the production of new protein agents. These spatial aspects of the agent-based model provide a greater level of detail and realism over more traditional forms of modelling, specifically in functional analysis of biological systems governed by spatial organisation.

The flexible agent-based supercomputing framework, FLAME (http://www.flame.ac.uk) and high performance computers were used to enable implementation and simulations at the scale required [15, 16]. In part, the model was developed using FLAME GPU, a version of the Flexible Largescale Agent-based Modelling Environment (http://www.flame.ac.uk) and modern Graphical Processing Units. For additional information on FLAME see S1 Text.

**Fig 1:**
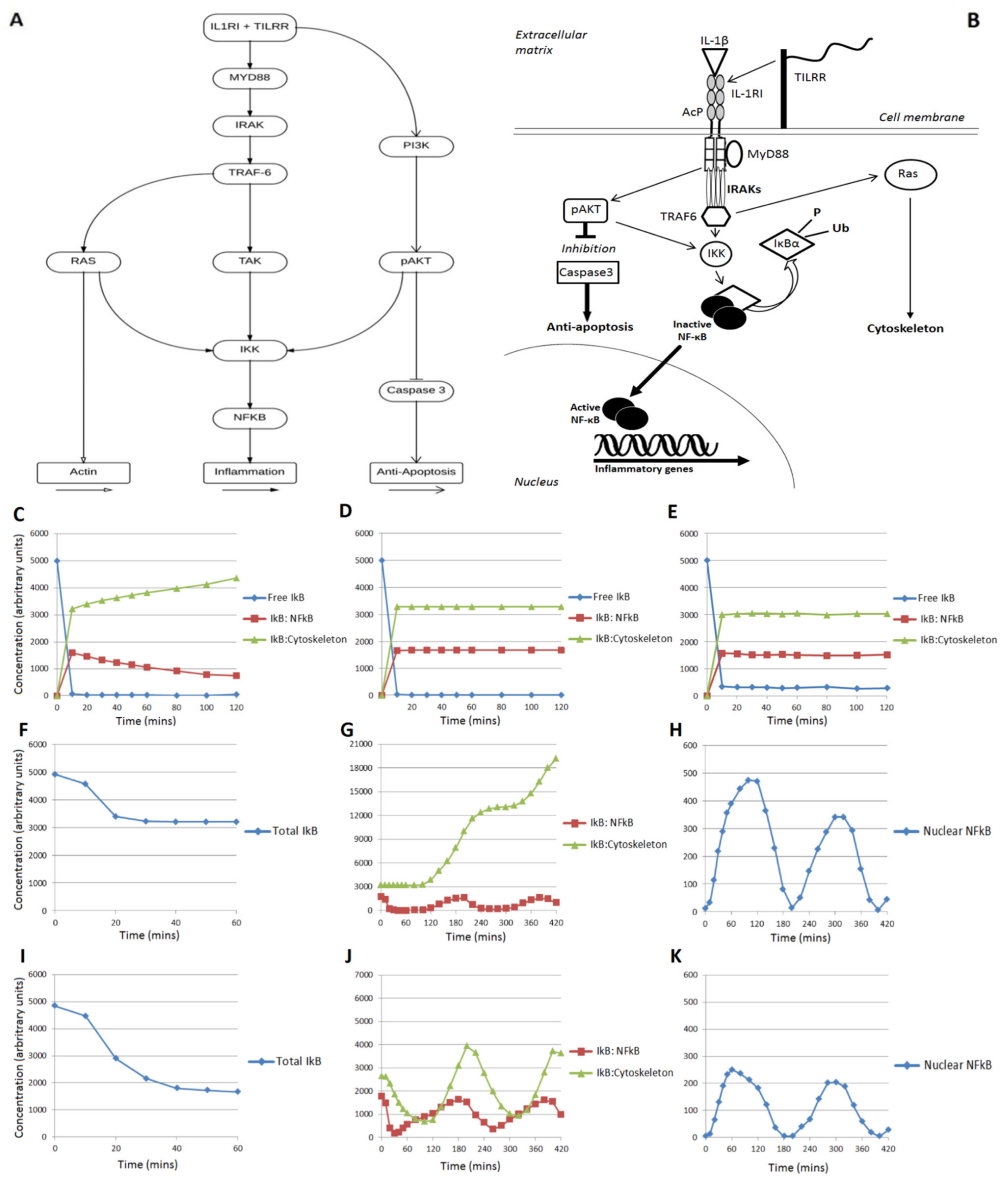
Agent based modelling including binding and release of cytoskeletal IκBα. A. Flowchart summarising the activation cascade and downstream effects incorporated into the agent based model of the NF-κB signalling pathway. B. Representation of localisation and movement of selected agents within the agent based model. C, D, E. Simulations of un-stimulated levels show accumulation of IκBα on the cytoskeleton with dissociation from NF-κB only (C) but a steady state when IκBα dissociates from neither (D) or both (E) cytoskeleton and NF-κB. F-H. Simulations of responses to IL-1 stimulation in the model with dissociation from neither cytoskeleton nor NF-κB. I-K Simulations of response to IL-1 stimulation in the model with dissociation from both cytoskeleton and NF-κB. Graphs show the average of 3 simulations for each condition. p<0.05 IκBα associated with the cytoskeleton at 120 min, C v. D, C v. E, p<0.005 IκBα levels at 60 min, F v. I.

The signalling pathway used in the model includes key proteins that control IL-1RI-induction of NF-κB triggered transcription (Fig 1A and 1B). It incorporates branching of signals leading to distinct effects to allow simulations and monitoring of how changes at specific steps propagate downstream through various aspects of the pathway.

Each simulation begins with an un-stimulated cell in a steady state, followed by IL-1β stimulation, induced by activating the IL-1RI agents and associated adaptor proteins, such as MyD88 (Myeloid differentiation primary response gene 88) or p85-PI3K (Phosphatidylinositol-4, 5-bisphosphate 3-kinase). The signal propagates and is amplified at each step of the pathway, and results in activation and nuclear translocation of NF-κB. Transcription site agents in the nucleus interact with NF-κB, triggering production of proteins including IκBα after a fixed transcription time. The new IκBα replaces the earlier degraded inhibitor and re-sequesters NF-κB to block further transcription. Therefore, continuous activation by IL-1β results in cyclic waves of stimulation reflecting IκBα degradation and transcription, and cessation of stimulation results in the cell returning to a steady state. Activation of the Akt kinase (protein kinase B) through PI3 kinase induces Caspase 3 through a non-canonical pathway, independent of IκBα degradation.

Parameters used in the simulations were adjusted to train the model to fit *in vitro* data and are presented in Table 1. To ensure the model was stable at a range of agent concentrations and not only functional at those chosen for the final model, a sensitivity analysis was performed. Simulations including concentrations ranging from 1/10 to 10-fold the optimal levels for each agent were analysed to confirm that the pathway was qualitatively performing as in the final model. In all cases the response to stimulations was reduced as agent numbers were varied, but the signal cascade and feedback response remained functional. To explore the differences in pathway activity resulting from IL-1β stimulation in the presence of WT TILRR or TILRR mutants, agent-based simulations were run under relevant conditions with the affinity of the receptor complex for its adaptor proteins adjusted, as described in Table 2.

**Table 1:** Agent Summary.

Name	Agent Type	Location	Potential States	Number of Agents
Nuclear Import	Receptor	Nuclear membrane	Active	2500
Nuclear Export	Receptor	Nuclear membrane	Active	200
IL-1RI + TILRR (WT or mutant)	Receptor	Cell membrane	Active, Inactive	Variable depending on experiment, up to 3000
IL-1RI	Receptor	Cell membrane	Active, Inactive	Variable depending on experiment, up to 3000
Cytoskeleton	Receptor	Cytoplasm	Active and unoccupied, Active and IκB bound, Inactive	600000
Transcription site	Receptor	Nucleus	Active	500
MyD88	Protein	Cytoplasm	Activated by TILRR, Activated by ACP, Inactive	20000
IRAK	Protein	Cytoplasm	Active, Inactive	20000
TRAF	Protein	Cytoplasm	Active, Inactive	20000
TAK	Protein	Cytoplasm	Active, Inactive	2000
Ras	Protein	Cytoplasm	Active, Inactive	20000
PI3k	Protein	Cytoplasm	Active, Inactive	20000
Akt	Protein	Cytoplasm	Active, Inactive	10000
IKK	Protein	Cytoplasm	Active, Inactive	20000
IκB	Protein	Cytoplasm, Nucleus	Free, bound to NFκB, bound to actin, being transcribed, to be degraded (pIκB)	Variable. Starting endogenous level 50000
NFκB	Protein	Cytoplasm, Nucleus	Free, bound to IκB	20000
IL-8	Protein	Cytoplasm, Nucleus	Active, being transcribed	Variable. Starting level 0
Caspase 3	Protein	Cytoplasm, Nucleus	Active, Inactive	2500
NFκB: IκB Dissociator	Protein	Cytoplasm	Active	500
Actin: IκB Dissociator	Protein	Cytoplasm	Active	70000
IκB Phosphorylator	Protein	Cytoplasm	Active	250

Summary of the agent types, location, potential states and starting numbers.

**Table 2:** Adapter-protein affinities for distinct IL-1RI complexes.

Receptor Complex	MyD88	pI3K
IL-1RI complex—TILRR	Low	Low
IL-1RI complex + WT TILRR	High	High
IL-1RI complex + D448 TILRR	Low	High
IL-1RI complex + R425 TILRR	High	Low

Summary of affinities for MyD88 and pI3K to the IL-1R1 receptor complex in the absence of TILRR, or in the presence of WT TILRR or TILRR mutants.

#### Agent Movement

The majority of the agents are free roaming, located within the cytoplasm or nucleus, while some are confined to specific locations within the cell or nuclear membranes. Free roaming agents move due to 3D Brownian motion and in a random direction during each iteration. In Brownian motion the mean square displacement of an individual particle is proportional to time and governed by a diffusion coefficient measured as length^2^/time. The diffusion coefficients were pre-set, using the same values for all agents in the model [17]. Instead of a fixed protein speed, a diffusion coefficient was used, as agent based modelling proceeds in discrete time steps, which can vary in length during iterations. The displacement of a protein is correlated with the step length, in a non-linear manner. By using the diffusion coefficient, the distance each protein moves in a given iteration does not need to be recalculated if the time step is changed, providing a more variable model. Transport between compartments requires transport receptors, in the absence of which the agent is retained. If an agent attempts to move beyond a compartment barrier such as the cell or nuclear wall, the agent is reflected back by the same distance it would have moved past the compartment wall, allowing it to travel the same distance but in a non-linear direction.

#### Agent Interactions

Agent-agent interactions, the core element of the model, are governed by strict rules. These determine which agents are able to interact and the effects of these interactions. The agents in the model have no physical size but instead interact with other agents within a predetermined radius. Due to the complexity of the signalling pathway, the model has been scaled down and uses interaction radii to maintain accurate reaction rates. Interaction radii allow agents to interact at a distance and are used to calibrate the rate of interactions at realistic concentration [18]. The reaction rate of the agent populations is determined by concentration, interaction radius and movement speed. Values for protein concentrations, speed and reaction rates were trained on available data to produce rates, which accurately reproduce the biological system. In cases of varying affinity such as IL-1RI recruitment of MyD88, (Table 2) the radius was adjusted, as required, to reflect the change.

#### 3D protein modelling and docking

The truncated tertiary structure of β-spectrin (NP_003119.2) was predicted using amino acid residues 27–749 from the N-terminal region to spectrin repeat (SR) 4. The spectrin 3D model was built using multiple-threading alignments and iterative fragment assembly in the de-novo I-Tasser Zhang Server [19]. Actin and IκBα were generated in Swiss-Model [20] using the template from the resolved structure complexes, PDB: 1YVN and PDB: 1IKN, respectively. Protein docking predictions and models were carried out using the generated PDB files in Gramm-X [21]. Protein tertiary structure models were viewed and modified in MolSoft ICM Browser [22]. C-score and TM-scores were calculated according to Zhang and Skolnick [23].

### Biological experiments

#### Transfection

HeLa cells were seeded (8x10^5^cells/10cm dish) and propagated in DMEM (Dulbecco's Modified Eagle Medium), (10% FCS, Foetal calf serum). At 24 hours cells were transfected with IκBα-EGFP (enhanced green fluorescent protein) (7μg) alone, or with ON-TARGETplus Non-targeting siRNA (10nM,Fisher), or custom TILRR siRNA (10nM, Eurogentec), using Polyfect (50μl; Qiagen Ltd), which as demonstrated in our earlier publication, causes a 60–80% knockdown of TILRR at mRNA and protein levels [13]. After further 24 hours, cultures were left un-stimulated or stimulated with IL-1β (1nM, R&D Systems) and IκBα phosphorylation (5, 10, 15, 30 minutes) and degradation (30, 60 minutes) determined.

#### Immunoprecipitation

Cells were cross-linked with DSS (7.5mM, 30°C, 50 min, Thermo Scientific) and reaction quenched (20mM TRIS, pH7.5, 15 min rt), and cells extracted in RIPA (500μl, 4°C, 30 min; Santa Cruz), aspirated through a 25-gauge needle, centrifuged at 17,000g (20 min) and protein levels determined by BSA assay (Pierce). The cytoskeletal fraction was immunoprecipitated with an anti-β-spectrin antibody (3μg/ml, 4°C, 3 hrs, Bethyl labs), or using an anti-actin antibody (3 μg/ml, 4°C, 3 hrs, Santa Cruz) and Sepharose G beads (50μl, 4°C, 2 hrs; GE Healthcare). Beads were washed 2x with Tris buffer (50mM, pH 7.6, 150 mM NaCl, 1% Triton-X) and 1x with PBS, and proteins eluted by heating in 1x reducing sample buffer (95°C, 7 min; Thermo Scientific).

#### Western Analysis

Immunoprecpitated samples were separated by SDS-PAGE (4–12%) and transferred to nitrocellulose membranes (GE Helathcare). Membranes were incubated with Milk (5%, Tris Buffered Saline Tween, TBST) and with an anti-IκBα antibody (1:1000; C21 rabbit polyclonal, O/N, 4°C, Santa Cruz), an anti-phospho-IκBα antibody (1/1000, Cell Signalling), an anti-spectrin antibody (1/1000) and with anti-β-actin (1/1000), washed in 1x TBST, incubated with IRDye 800CW Donkey anti-Rabbit (1:10,000, 1 hr; LI-COR Biosciences), washed, and developed using LI-COR Odyssey. The intensity of a 135kDa band, constituting the 80kDa spectrin and the IκBα-EGFP, was determined using ImageJ (Version 1.66f or J64), as previously [13,14].

Pre-immunoprecipitation samples (input) were analysed as above and input levels of spectrin, actin, IκBα and IκBα phosphorylation determined for all conditions. Additional controls included samples immunoprecipitated with a non-specific IgG, which showed no signal.

#### Statistical Analysis

Statistical analysis used GraphPad Prism and two-tailed unpaired t-tests with Welch’s correction to calculate p-values. Significance of data from biological experiments was calculated using the mean ±sd of 3–5 experiments, for each condition, as indicated in figure legends. *In silico* simulations were run 3–4 times, and mean±sd for each condition and time-point was compared with the relevant control simulation output.

## Results and Discussion

### Agent Based Modelling of cytoskeletal binding and release of IκBα

Our earlier work used a combination of *in silico* and *in vitro* approaches to demonstrate that a significant proportion of IκBα is bound to the cytoskeleton in the resting cell [11]. To assess the role of the cytoskeletal pool of the inhibitor in NF-κB control, the original model was expanded to include regulators of the canonical and non-canonical NF-κB pathways, the IL-1 receptor complex including the co-receptor TILRR, and components of the cytoskeleton (Fig 1A and 1B). Throughout development of the model predictions were validated by data from *in vitro* experiments, which included real time recordings of signalling events in live cells, in order to ascertain an accurate reproduction of the biological system.

Simulations to test the impact of the cytoskeletal pool of the inhibitor show that in the absence of dissociation, the system fails to reach a steady state under resting conditions (Fig 1C). Instead IκBα is successively recruited to the cytoskeleton, following dissociation from NF-κB. Further, the analysis demonstrated that the un-stimulated system reaches a steady state under conditions where IκBα dissociates from neither the cytoskeleton nor NF-κB (Fig 1D), or when the inhibitor is able to dissociate from both (Fig 1E).

Subsequent analysis compared IL-1-induced activation under conditions used in Fig 1D and 1E. Simulations showed that in the absence of IκBα-dissociation (as in D), inhibitor degradation is reduced by about 50% compared to levels recorded in *in vitro* experiments, with an increasing proportion of IκBα successively recruited to the cytoskeleton (Fig 1F, 1G and 1H). In contrast, in the presence of dissociation of IκBα from both NF-κB and the cytoskeleton (as in E) the degradation profile fits the biological data (Fig 1I) [24]. Further, simulations over 6 hours stimulation using these settings, demonstrated the expected IκBα distribution and nuclear NF-κB translocation, reflecting activation and negative feedback (Fig 1J and 1K).

### Three-dimensional reconstruction of IκBα interaction with the actin/β-spectrin complex

**Fig 2:**
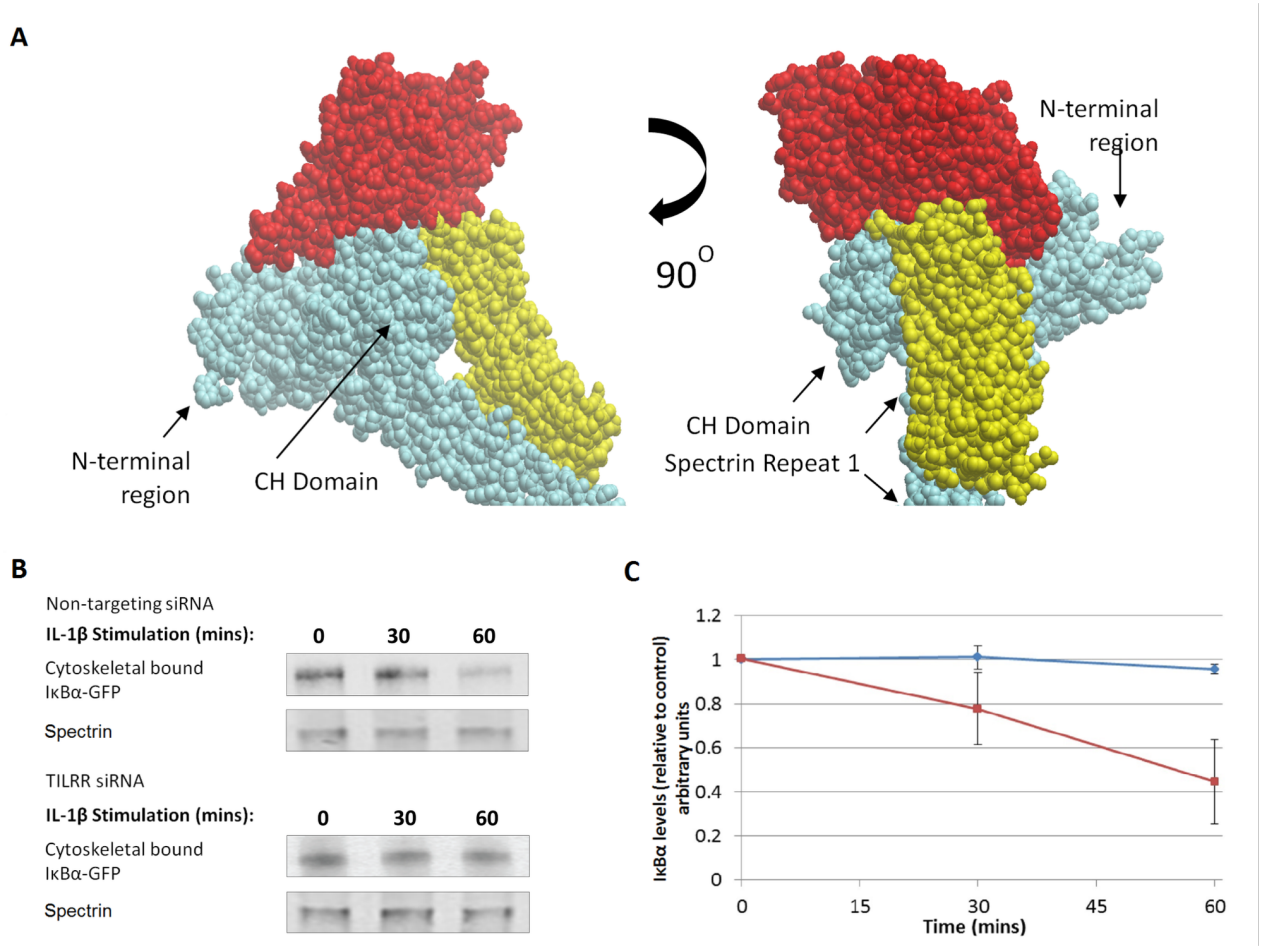
IκBα interaction with cytoskeletal proteins. A. Space-filling representation of the predicted binding interaction of IκBα with cytoskeletal proteins spectrin and actin. Two orientations of the complex are shown to illustrate binding interactions between the three molecules. β-spectrin is shown in blue, actin in red and IκBα in yellow. B. HeLa cells were transfected with IκBα-EGFP together with a non-targeting siRNA or TILRR siRNA. Cell lysates were immunoprecipitated with an anti-spectrin antibody, and levels of spectrin and of spectrin-associated IκBα determined by immunoblotting, as described in material and methods. C. Quantitation of spectrin associated IκBα-EGFP from western blots of cells transfected with random siRNA (control) or TILRR siRNA, and stimulated with IL-1β (1nM) over time, as in B. Data are presented as fraction of levels in un-stimulated samples (t = 0) and expressed relative to loading control (Mean ±SEM, n = 5, p<0.05 at 60 min).

Initial analysis was designed to determine the predicted protein-protein interactions within the actin/β-spectrin complex, followed by a second step to assess IκBα binding to the cytoskeletal components. The structure and conformation of β-spectrin, from N-terminal to Spectrin Repeat (SR) 4, was predicted using resolved crystal structures of SRs, CH domain and α-actin (PDB: 1CUN, 1U5P, 3FB2, and 1S35, 1AA2, 1SJJ) as the major templates for de-novo assembly of the truncated β-spectrin molecule. Verification of the actin/spectrin interaction model showed high similarity to recently published homology modelling of the mini-spectrin tetramer [12]. A calculated C-score of -1.46 demonstrated a medium-high confidence for the quality of the predicted model, and a TM-score of 0.53±0.15 confirmed correct topology [23]. Protein docking analysis predicted, as expected, actin binding within the CH domain of β-spectrin. Subsequent, analysis to assess the binding of IκBα showed association of the inhibitor with repeat 1 of β-spectrin (SR1) and bridging the CH domain (Fig 2A, S1 Fig). In addition, the model demonstrates binding of the inhibitor to actin, which is consistent with our earlier findings and could reflect interactions through ankyrin binding sites [11, 25–27].

Cytoskeletal binding of IκBα was confirmed by *in vitro* experiments, using cross-linking and immunoprecipitation based on association with spectrin and actin (Fig 2B, S2 Fig). In addition, these experiments demonstrated a successive reduction in the cytoskeletal pool of the inhibitor during pathway activation. Quantitation showed an average decrease in IκBα levels of 20% after 30 minutes of IL-1 stimulation, with a further reduction to levels corresponding to about 45% after 60 minutes (Fig 2C, S2 Fig). Similarly, inhibitor levels in pre-immunoprecipitation samples (input) were successively reduced during IL-1 stimulation (S3 Fig). In contrast, levels of spectrin and actin in both input and immunoprecipitated samples were unaltered by any of the conditions used (Fig 2B, S3 Fig). Further analysis demonstrated the expected peak of phosphorylation in the input after 5 minutes of IL-1 stimulation, while the cytoskeletal-associated inhibitor remained un-phosphorylated throughout the time course (data not shown). The data are consistent with a mechanism involving successive release of un-phosphorylated inhibitor from the cytoskeletal pool during activation-induced degradation of cytoplasmic IκBα.

The ligand-induced release in both input and immunoprecpitated samples was abrogated in the presence of TILRR specific siRNA, in agreement with our published data demonstrating a role for the IL-1RI co-receptor in IL-1-induced IκBα degradation (Fig 2C) [13]. Further, the results are consistent with TILRR control of cell adhesion and the cytoskeleton, and of regulation of Ras (Rat sarcoma small GTPase) dependent amplification of NF-κB [13].

### Predictive modelling of release of cytoskeletal bound IκBα

**Fig 3:**
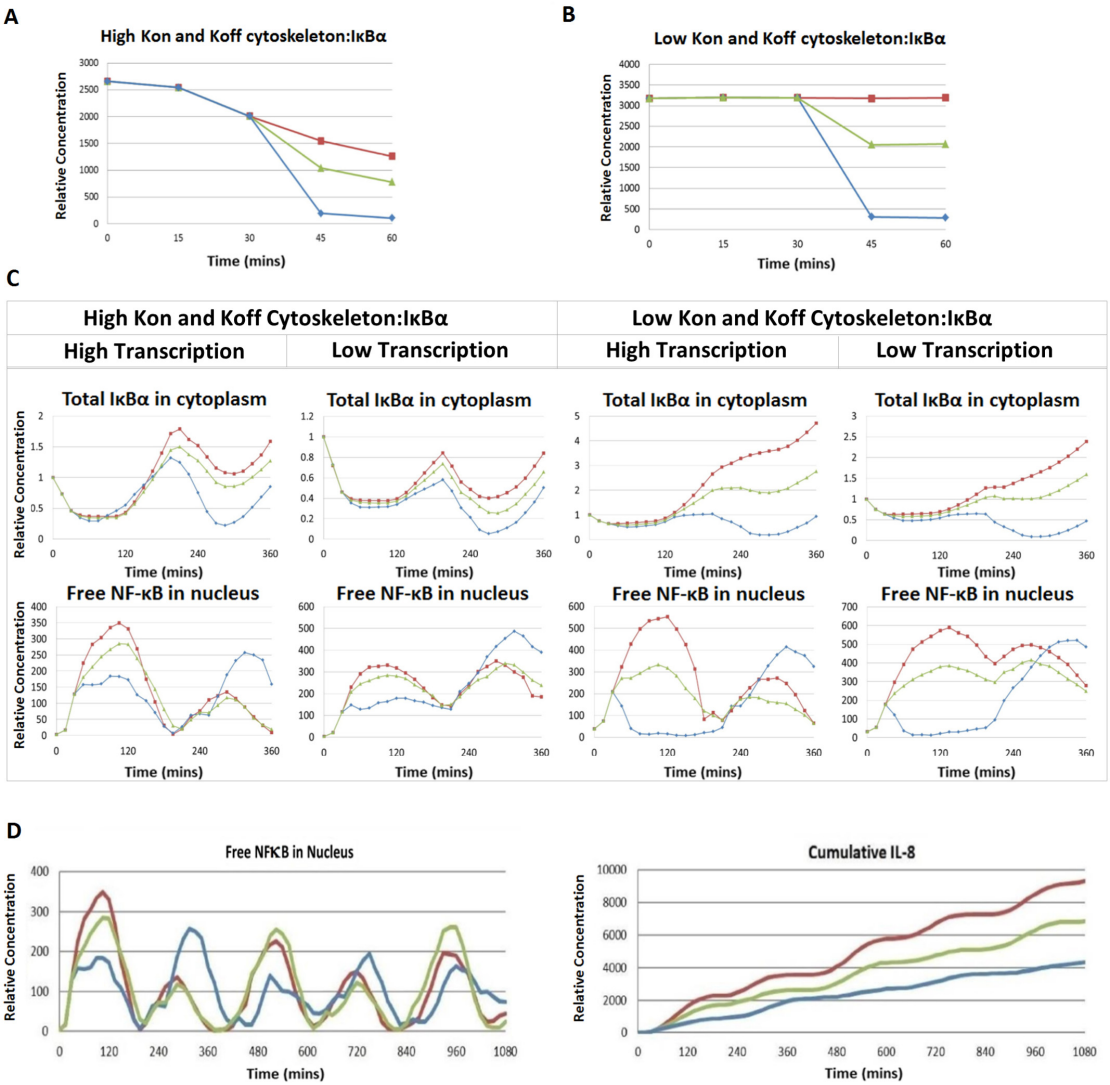
IL-1-induced release of IκBα from the cytoskeleton is characterized by high Kon /Koff rates. A, B. Simulation of levels of IκBα bound to the cytoskeleton during IL-1β stimulation under various settings. Release from the cytoskeleton was governed by Kon and Koff rates, adjusted to be high (A) or low (B) at the same Ka, with 2/3 of IκBα bound to the cytoskeleton at time = 0. Inhibitor release was set at 3 levels passive release only (Red), or release of 40–50% (Green) or of 90–100% (Blue). The model including high Kon and Koff rates fits best with *in vitro* data seen in Fig 2C. A. p<0.05, passive vs 40–50% release, p<0.005 passive vs 90–100% release. B. p<0.0005 passive vs 40–50% and 90–100% release. C. Predicted levels of IκBα and nuclear NF-κB using simulation settings in A and B at 3 levels of release as in A and B, and comparing two levels of transcriptional response. Simulations using high Kon and Koff rates and high transcription rates result in a behaviour that fits the *in vitro* data. D. Simulation output over 18 hours using the best-fit model (high Kon and Koff rates, high transcription rates) and 3 levels of inhibitor release, as in A and C, show nuclear NF-κB (left graph) and IL-8 gene activity (right graph). Simulations predict that all 3 levels of IκBα release are functional over multiple cycles of nuclear translocation (left), and prolonged inflammatory responses (right). IL-8 accumulation at 1080 min p<0.005 passive vs 40–50% release, p<0.0005 passive vs. 90–100% release. Graphs in A-D show an average of 3 simulations for each condition.

Based on these results, further validations used the expanded model, including dissociation of IκBα from the cytoskeletal pool during IL-1 stimulation shown in Fig 1I, 1J and 1K. These *in silico* simulations initially tested a range of parameters to determine the characteristics of the cytoskeleton:IκBα binding, which included high and low exchange rates (Kon and Koff) and 3 levels of dissociation (passive release only; passive plus 40–50% active release; passive plus 90–100% active release). Simulations using the fast exchange model (high Kon /Koff) predicted a successive reduction in the level of IκBα bound to the cytoskeleton at all three rates of dissociation, in good agreement with the *in vitro* data (Figs 2C and 3A). In contrast, simulations using the slow exchange model showed a negligible passive release, and predicted a 30-minute lag time, followed by a 15-minute rapid dissociation at 40% and 90% active inhibitor release (Fig 3B). Further analysis confirmed a good fit between the fast exchange model and the *in vitro* data at three levels of IκBα dissociation, and predicted the increased inhibitor release to reduce NF-κB nuclear translocation during the initial stimulation cycle, as expected (Fig 3C). Instead, the slow exchange model (low Kon and Koff) did not agree with the *in vitro* data, as it predicted a continuous rise in inhibitor levels during stimulation, and was therefore rejected.

Further analysis used the fast exchange model to simulate IL-1-induced NF-κB nuclear translocation and gene activity over longer stimulation times (Fig 3D). Results showed that, while the initial peaks were affected by IκBα release, the frequency and amplitude of subsequent oscillations were largely independent of inhibitor levels. In the presence of passive IκBα release or 40–50% active dissociation, the model predicted the second peak of activity to be significantly reduced, consistent with transcriptional induction of IκBα. In contrast, under conditions of 90–100% release, the second activation peak was much enhanced, reflecting a low initial induction of IκBα. Further simulations, which used IL-8 induction as readout, predicted that all 3 levels of IκBα release are functional at the level of gene activity during prolonged inflammatory responses (Fig 3D).

The model predicts that binding of IκBα to the cytoskeleton is transient and that IL-1 stimulation and degradation of the cytoplasmic inhibitor lead to its partial release. Further, the data suggest dissociation of the cytoskeletal pool of IκBα to provide a mechanism for rapid negative feedback during activation of NF-κB. Subsequent *in silico* experiments, used the fast exchange model with 40–50% release, combined with a high transcription rate, which showed the best fit with the *in vitro* data. Additional details on this model are included in S1–S5 Texts, S1–S3 Tables.

### The model accurately reproduces biological data on IκBα degradation and gene activity

**Fig 4:**
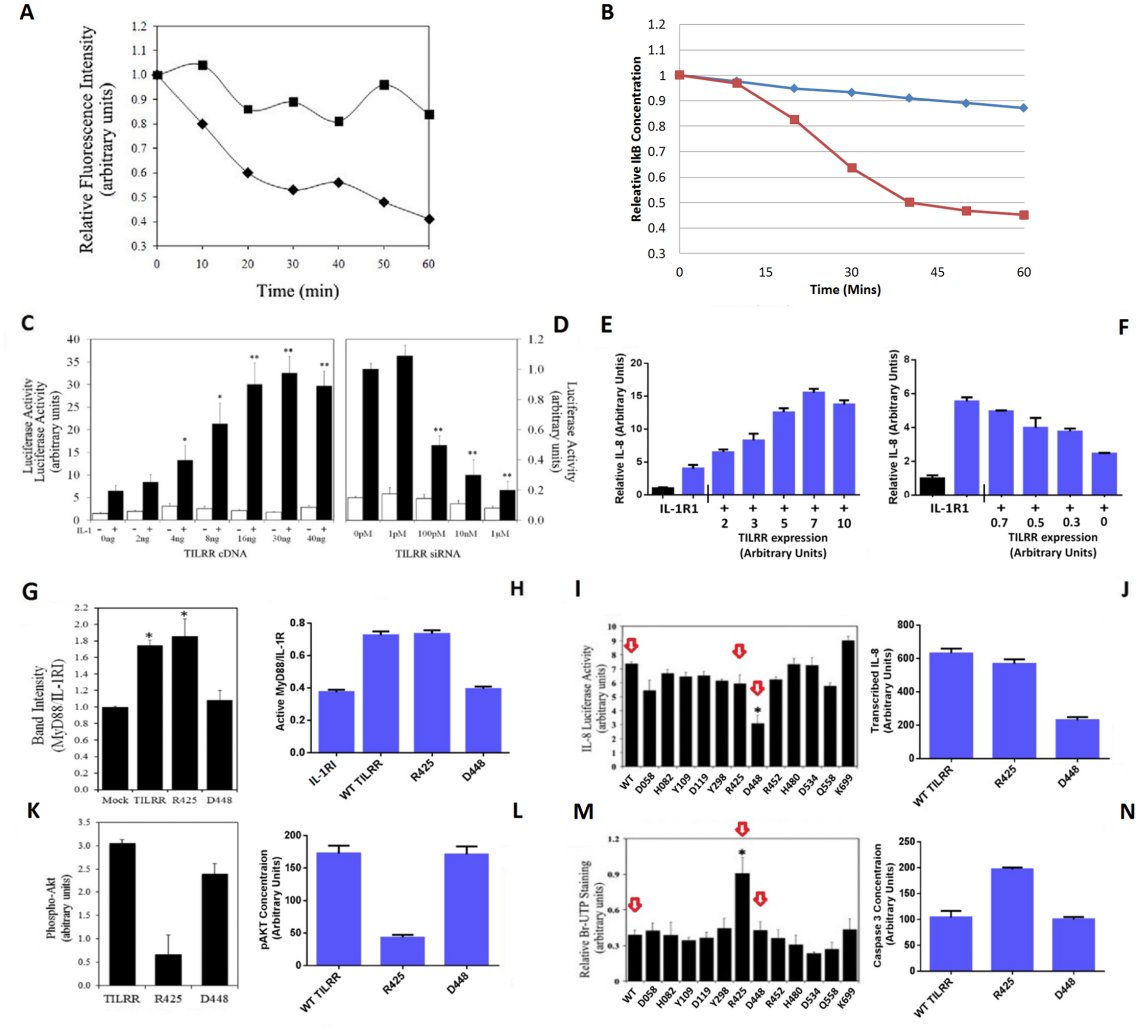
The model accurately reproduces activation of inflammatory and anti-apoptotic signals, controlled through IL-1RI and TILRR. A. TILRR siRNA decreases IκBα degradation in HeLa cells transfected with IκBα-EGFP (7μg/10^6^ cells) alone or in the presence of random (♦) or TILRR-specific (■) siRNA and stimulated with IL-1 (1 nM). Graph show continuous readings from three experiments including 55 cells, p<0.05 at 60 min. B. Simulations using the best-fit model, show reductions in transfected IκBα-EGFP degradation during IL-1β stimulation in the presence (Red) and absence (Blue) of TILRR, and are in agreement with published data (A). C, D. TILRR cDNA increases (C) and TILRR siRNA decreases (D) IL-8 luciferase activity in HeLa cells treated with IL-1β (1nM, 6 hrs.) (Mean±SEM, n = 3, *p <0.05; **p< 0.01). E, F. Simulations of un-stimulated levels (black) and IL-1β-induced IL-8 activity (blue) in the presence of the IL-1RI complex and with increasing (E) and decreasing (F) TILRR expression relative to endogenous levels, using the agent-based model, show agreement with *in vitro* data in C and D. (Mean±SEM, n = 3, 0.005<p<0.03, E. TILRR expression levels 2–10, F. TILRR expression 0.3, 0). G, H. MyD88 recruitment to the IL-1RI complex during IL-1β stimulation (20 min, 1nM), is enhanced following transfection with wild type (WT) TILRR or the R425 TILRR mutant, while substitution at residue D448, abrogates the effect. *In vitro* experiments (G) (Mean± SEM, n = 3, * p <0.005) and model simulation (H) (Mean ± SEM, n = 3, WT vs. D448 p<0.0.001). I, J. Mutation of residue D448 results in a 60% reduction in inflammatory gene activity, while other mutants have no impact (I) (arrows indicate WT TILRR and the R425 and D448 mutants, Mean±SEM, n = 3, * p<0.001). Model simulations (J) similarly show a pronounced reduction in the presence of the D448 mutant, but no effect by substitution of R425. (Mean±SEM, n = 3, WT vs. D448 p<0.001). K, L. TILRR-induced increase in levels of phosphor-Akt by IL-1β (1nM) is blocked following mutation of residue R425, while substitution at D448 has the same effect as the wild type co-receptor. *In vitro* experiments (K) (Data are expressed relative to levels in mock transfected cells, Mean±SEM, n = 3, WT vs. R425, p<0.01), and model simulation (L) (Mean±SEM, n = 3, wt vs. R425, p<0.005). M, N. Mutation of TILRR residue R425 blocks anti-apoptotic responses, determined by TUNEL staining in *in vitro* experiments, while the D448 substitution has no impact (M) (Mean ±SEM, n = 3, * p<0.001.). Model simulations show that TILRR-induced reductions in pro-apoptotic Caspase-3, is blocked by the R425 mutation but unaffected by substitution at D448 (N) (Mean±SEM, n = 3, wt vs. R425, p<0.01). Graphs in A, C, D, G, I, K, M are reproduced from Zhang, *et al*. [13,14], and used for validation of the model and for comparison with agent based model output.

Simulations faithfully reproduced kinetics and levels of IκBα degradation obtained by real time measurements, confirming a high level of accuracy, as single cell readings provide a sensitive readout of NF-κB activity (Fig 4A and 4B) [24, 28–30]. Validation of the model output at the level of gene activity, similarly demonstrated an accurate representation of IL-1-induced responses, and showed a successive increase in activation with higher TILRR expression levels, in agreement with *in vitro* experiments (Fig 4C, 4D, 4E and 4F) [13,14]. Further expansion of the model will make it possible to increase the complexity of the simulations and consider additional parameters such as the impact of other IκB isoforms and signalling receptor crosstalk on this process.

### The model reproduces distinct effects on downstream signalling, induced by TILRR mutants

To compare the impact of the cytoskeletal release on inflammatory and anti-apoptotic signals, the model was designed to allow selective regulation of MyD88 dependent and Akt-controlled responses, induced through PI3 kinase. The distinct pathways were modelled using *in vitro* data from experiments, which included TILRR mutants with distinct effects on signalling responses to IL-1β [14]. While alanine substitution of residue D448 inhibits amplification of MyD88-dependent inflammatory responses, it has no impact on Akt-regulated anti-apoptotic signals (Fig 4G, 4I, 4K and 4M). Conversely mutation of residue R425 does not affect inflammatory responses, but blocks Akt-phosphorylation and anti-apoptotic signals.

Model simulations used identical starting conditions, and including the IL-1RI complex in the absence of TILRR, or in the presence of wild type (WT) TILRR, D448A TILRR or R425A TILRR. Affinities of the various receptor complexes to the adapter proteins were based on *in vitro* data and are listed in Table 2. Results show that changes in adapter affinity and recruitment at the level of the receptor, propagate through the signalling pathway and selectively impact inflammatory and anti-apoptotic responses as seen *in vitro* (Fig 4G, 4H, 4I, 4J, 4K, 4L, 4M and 4N). Similarly, *in silico* simulations of IκBα degradation under the control of wild type and mutant TILRR, show good agreement with *in vitro* data, obtained from single cell readings (S4 Fig). The highest level of inhibitor degradation is induced in the presence of WT TILRR. Substitution of residue R425 has little effect on IκBα turnover, while mutation of residue D448 significantly impairs inhibitor degradation. Results are consistent with IκBα degradation-dependent control of inflammatory activation through MyD88, and regulation of Akt-induced anti-apoptotic responses by an IκBα independent mechanism, through PI3 kinase [31].

### Model simulations predict a role for cytoskeletal binding and release of IκBα in regulation of NF-κB inflammatory signal amplification and gene activation

**Fig 5:**
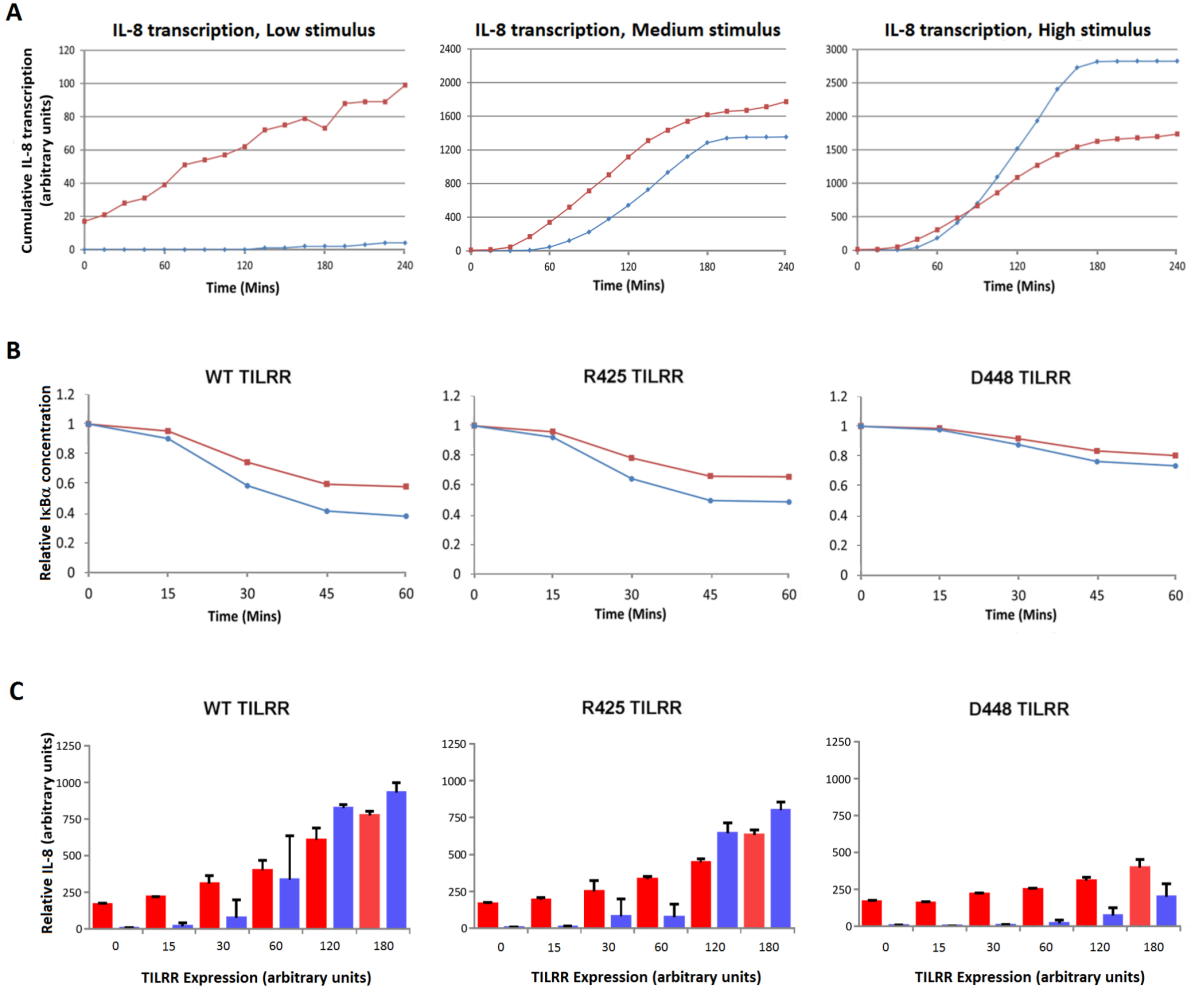
Cytoskeletal binding and release of IκBα controls NF-κB signalling and gene induction. A. Simulated IL-8 gene activity kinetics at varying IL-1 pathway stimulation levels, in the presence (Red) and absence (Blue) of cytoskeletal binding and release of IκBα. Simulations show that disabling cytoskeletal binding of the inhibitor abrogates activation at low levels of stimulation, causes delayed responses at medium stimulation, and results in a slightly delayed but significantly increased activation at high levels of stimulus. P < 0.05 at low and high stimulus. B. Simulated degradation of IκBα after stimulation with saturated levels of IL-1β (10^-9^M) with enabled (Red) or disabled (Blue) cytoskeletal binding and release of IκBα, in the presence WT TILRR, R425 TILRR or D448 TILRR. The model predicts that cytoskeletal binding of IκBα causes a reduction in its degradation in the presence of WT TILRR and both mutants, consistent with a greater IL-8 response at higher levels of stimulation. The low level of IκBα-degradation induced in the presence of the 448 mutant is similarly affected by cytoskeletal binding of the inhibitor. n = 4 p>0.05 at 60 min for all conditions. C. Predicted IL-8 activity at varying TILRR expression levels, with enabled (Red) or disabled (Blue) cytoskeleton: IκBα binding, and in the presence of WT TILRR, or TILRR mutants R425 or D448. As expected, inflammatory activity is higher in the presence of wild type TILRR or the 425 mutant. Comparing activity in the presence and absence of cytoskeletal binding, under these conditions, show a more pronounced effect at lower levels with inverse effects at higher levels of, activity, while effects at intermediate activities are less pronounced consistent with data in 5A. Levels in the presence of the 448 mutants correspond to the lower range of activity, only. (Mean±SEM, n = 3). WT, 425 0.0005< p< 0.05 at expression levels 0, 15, 120, 180, ns 30, 60. D448. 0.0005< p < 0.05 at expression levels 0–180.

To determine the effect of cytoskeletal sequestration and release of IκBα on receptor induced activation, we disabled the process and compared activation at three stimulus levels, using the inflammatory gene IL-8 as readout (Fig 5A). Simulations predict low levels of stimulation to be most sensitive to cytoskeletal release of the inhibitor, and activation to be abrogated in the absence of cytoskeleton: IκBα binding. At intermediate levels, lack of cytoskeletal binding of the inhibitor increases lag time and results in a moderate reduction in gene activity. At high stimulation, disabling the process causes an increased lag time, followed by pronounced signal-amplification and enhanced activation levels, which by 90 minutes exceed responses in the presence cytoskeletal binding and release of IκBα.

Subsequent simulations were designed to determine the impact of the cytoskeleton on MyD88-controlled inflammatory responses and Akt-induced anti-apoptotic signals. Selective activation of the pathways was validated by comparing model outputs with *in vitro* experiments including the TILRR mutants, as in Fig 4G, 4H, 4I, 4J, 4K, 4L, 4M and 4N [14]. Results from initial simulations showed that sequestration to the cytoskeleton caused significant reductions in IκBα degradation during IL-1 stimulation under all three conditions. The rate and level of inhibitor degradation were similar in the presence of wild type TILRR and the R425 mutant, which both activate IκBα degradation dependent responses. In contrast, as expected, in the presence of the D448-mutant, which blocks 60% of MyD88-dependent signals, IκBα degradation was much reduced (Fig 5B).

The effects of the cytoskeleton at the level of gene regulation, was assessed using a range of expression levels of WT TILRR or TILRR mutants (Fig 5C). The model predicts that in the presence of wild type TILRR and the 425 mutant, cytoskeletal binding and release of IκBα increases gene induction at low stimulus, and reduces activity at higher levels of TILRR and increased signal amplification, in agreement with data in Fig 5A. As expected, at the low level of IκBα degradation dependent signals induced in the presence of the D448 mutant, cytoskeletal sequestering and release significantly increased activity over the same concentration range (Fig 4G, 4H, 4I and 4J) [13,14]. In contrast, Akt-controlled anti-apoptotic signals, which were the same in the presence of wild type TILRR and the D448 mutant and reduced by the R425 substitution, were unaffected by cytoskeletal binding of the inhibitor under all conditions. The results are consistent with a distinct impact of cytoskeletal sequestration and release of inhibitor, on IκBα-degradation dependent control of inflammatory responses.

The simulations show that in the absence of cytoskeletal binding, there is a threshold of input activity required to produce an IκBα-degradation dependent inflammatory response. Once this level of stimulation is reached, the response rapidly becomes maximal in a concentration dependent manner. The model predicts that cytoskeletal sequestration and stimulation-induced release of IκBα, contribute to control of NF-κB by modifying pathway activation in relation to input levels. Binding of IκBα at low-level stimulations allows signal propagation despite a high cellular IκBα/NF-κB ratio, while release of the inhibitor at higher levels of stimulation induces a rapid negative feedback.

The mechanism provides input-sensitive control of IL-1- induced signal amplification, and allows early calibration of NF-κB activity and inflammatory responses. The process is expected to be relevant to regulation of host defence mechanisms as well as to dys-regulation of NF-κB activation in disease [2, 9, 10].

## Conclusion

The cytoskeleton controls NF-κB activation of inflammatory responses through signal-dependent sequestration and release of the inhibitor IκBα.

## Supporting Information

S1 Fig 3-Dimensional space-filling representation of predicted binding interaction between IκBα and cytoskeletal proteins, spectrin and actin. Interactions predicted by 3D model of spectrin (SR1-4), actin and IκBα using de-novo threading modelling, comparative modelling and an iterative protein docking approach.
(PDF)

S2 Fig IκBα association with the actin cytoskeleton. Association of IκBα with the actin cytoskeleton and successive reduction of the actin bound inhibitor during IL-1 stimulation.
(PDF)

S3 Fig Western analysis of IκBα and spectrin levels in pre-immunoprecipitation samples (input). Time dependent reduction in IκBα levels during IL-1 stimulation, which is reduced in the presence of TILRR siRNA. Spectrin levels are unaltered.
(PDF)

S4 Fig *In silico* simulations accurately reproduce data from *in vitro* experiments showing varying degrees of IκBα degradation induced by TILRR mutants. *In vitro* data obtained by single cell readings from live cells are compared with *in silico* simulations.
(PDF)

S5 Fig Effects of cytoskeletal binding and release of IκBα on levels of active Caspase 3. Simulations comparing effects of cytoskeletal binding in the presence of wild type and mutant TILRR show no effect under any of the conditions.
(PDF)

S1 Table Protein agent memory variables. The Protein agent uses eight memory variables to record agent type, location and state.
(PDF)

S2 Table Protein agent messages. Agent communications in FLAME are messages sent and received by agents. Messages from Protein agents signal their location and state, as well as requests for interactions.
(PDF)

S3 Table Protein agent functions. Agent functions are performed in a predefined order, and include outputting messages, reading messages and updating the internal state.
(PDF)

S1 Text The Agent-Based Modelling Framework: FLAME. FLAME is an agent-based modelling framework designed for high performance and parallel processing of agents.
(PDF)

S2 Text Agent Based Modelling. The FLAME agent based model is developed in three steps—agent’s memory and functions, implementation of functions, and simulation of the starting state. Receptors and signalling intermediates are represented as agents and simulations describe key signalling events.
(PDF)

S3 Text Agent Name: Protein. Free roaming proteins involved in the signal pathway act according to defined rules for Protein functions, determined by the type and state of the Protein agent.
(PDF)

S4 Text Steady State. The initial state of the agents is set to an inactive form followed by two hours simulations of the un-stimulated steady state, prior to simulating activation through the IL-1 type I receptor.
(PDF)

S5 Text Agent Numbers and Affinities. Reaction rates are determined by the concentration of reagents and their affinity.
(PDF)
