# Supplementary material for: Computational Modelling of NF-κB Activation by IL-1RI and Its Co-Receptor TILRR, Predicts a Role for Cytoskeletal Sequestration of IκBα in Inflammatory Signalling
Source: PLoS One. 2015 Jun 25;10(6):e0129888. doi: 10.1371/journal.pone.0129888 (PMC4482363; doi:10.1371/journal.pone.0129888)
Supplement: S1 Fig — Interactions predicted by 3D model of spectrin (SR1-4), actin and IκBα using de-novo threading modelling, comparative modelling and an iterative protein docking approach. (PDF) [file pone.0129888.s001.pdf]

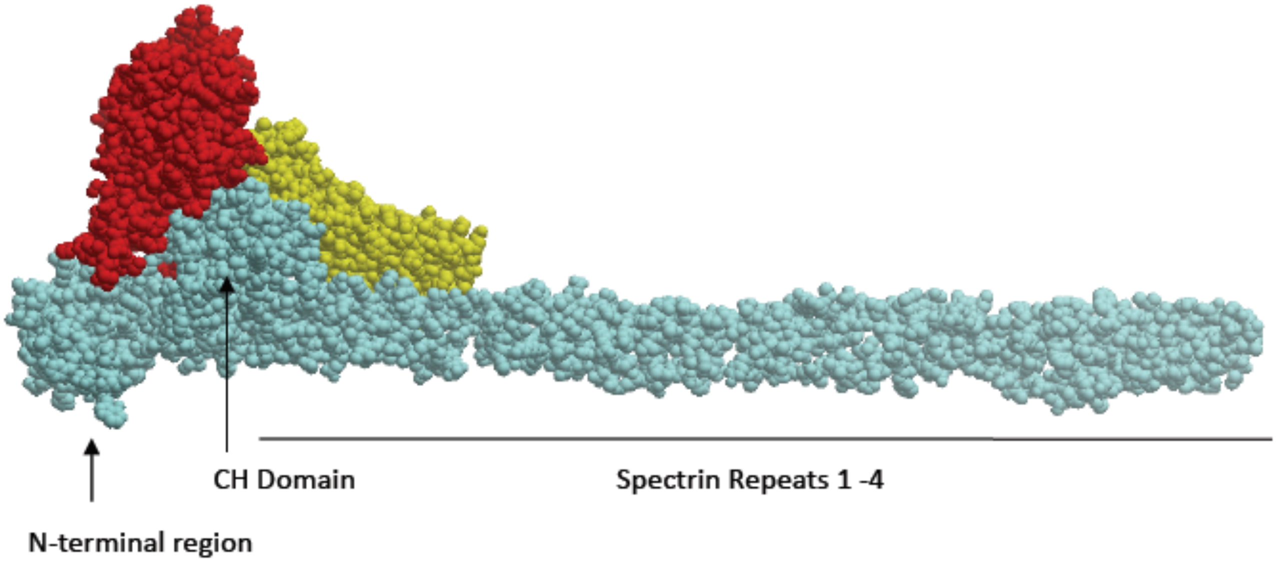

S1 Fig. 3-dimensional space-filling representation of predicted binding interaction between IκBα and cytoskeletal proteins, spectrin and actin.
The model was generated using de-novo modelling and multiple-threading alignments together with iterative fragment assembly in the de-novo I-Tasser Zhang Server. The truncated tertiary structure of β-spectrin (NP_003119.2) was predicted using amino acid residues 27 – 749 from the N-terminal region to spectrin repeat (SR) 4. Actin and IκBα were generated in Swiss-Model using the template from the resolved structure complexes, PDB: 1YVN and PDB: 1IKN, respectively. Protein docking using generated PDB files were generated in Gramm-X and modified in MolSoft ICM Browser, as described in Material and Methods (19-22). The analysis shows binding of IκBα to the spectrin 1 repeat (SR1) and confirms interaction with actin associating in the region of the CH domain. Bound β-spectrin, including repeats 1-4, is shown in blue, actin in red and IκBα in yellow.
