# Supplementary material for: Computational Modelling of NF-κB Activation by IL-1RI and Its Co-Receptor TILRR, Predicts a Role for Cytoskeletal Sequestration of IκBα in Inflammatory Signalling
Source: PLoS One. 2015 Jun 25;10(6):e0129888. doi: 10.1371/journal.pone.0129888 (PMC4482363; doi:10.1371/journal.pone.0129888)
Supplement: S2 Fig — Association of IκBα with the actin cytoskeleton and successive reduction of the actin bound inhibitor during IL-1 stimulation. (PDF) [file pone.0129888.s002.pdf]

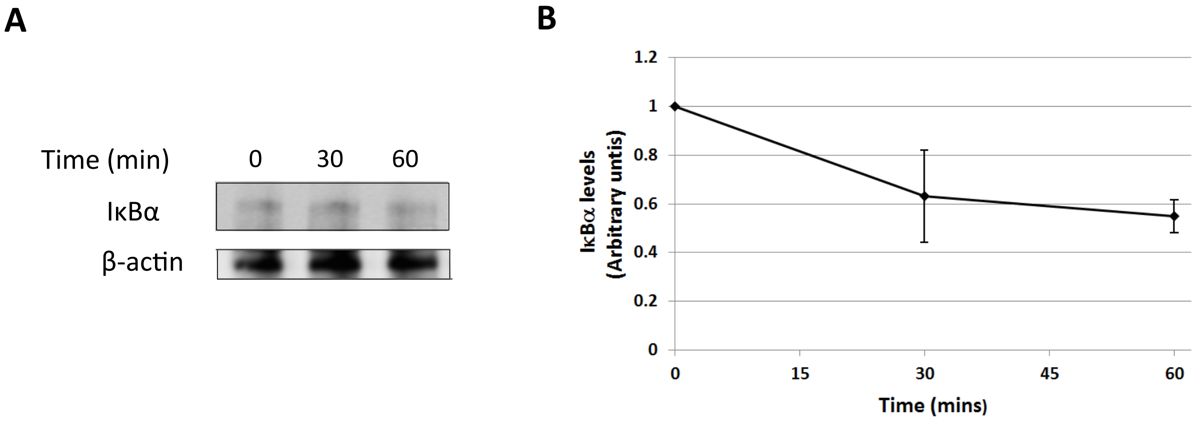

S2 Fig. IκBα association with the actin cytoskeleton

Cells were stimulated with IL-1 (10^{-9}M) for the times indicated, samples crosslinked, and immunoprecipitated using anti-alpha actin and levels of actin associated IκBα determined by western blotting (A). Quantitation shows a successive reduction in actin associated IκBα corresponding to about 50% over 60 minutes, comparable to the profile obtained following spectrin pull down shown in Figure 2 (B). n=3 $p<0.005$ at 60 min.
