# Supplementary material for: Computational Modelling of NF-κB Activation by IL-1RI and Its Co-Receptor TILRR, Predicts a Role for Cytoskeletal Sequestration of IκBα in Inflammatory Signalling
Source: PLoS One. 2015 Jun 25;10(6):e0129888. doi: 10.1371/journal.pone.0129888 (PMC4482363; doi:10.1371/journal.pone.0129888)
Supplement: S3 Fig — Time dependent reduction in IκBα levels during IL-1 stimulation, which is reduced in the presence of TILRR siRNA. Spectrin levels are unaltered. (PDF) [file pone.0129888.s003.pdf]

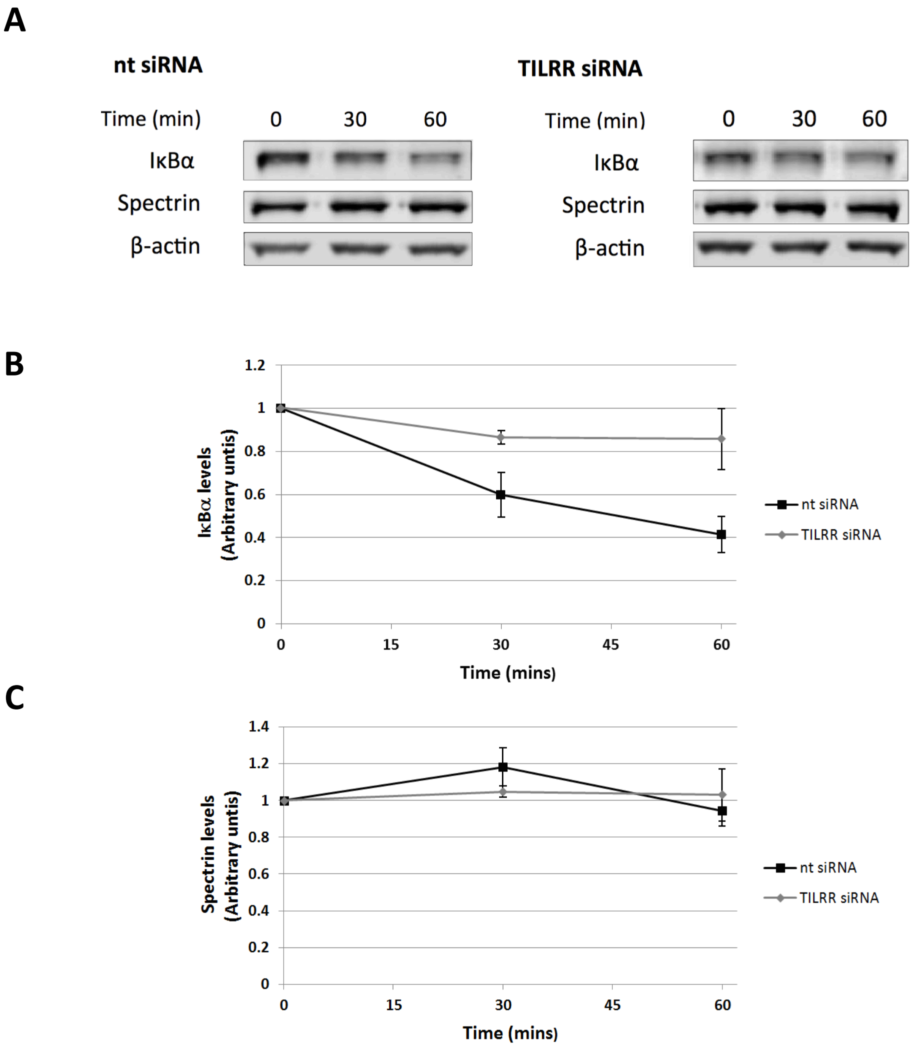

S3 Fig. Pre-immunoprecipitation levels of IκBα and spectrin.
Cells were stimulated with IL-1 (10^{-9}M) for the times indicated and samples crosslinked, as described in Material and Methods, and levels of IκBα and spectrin determined for the various conditions by Western blotting (A). Quantitation shows a successive reduction in IκBα levels by 60% in the presence of non-targeting (nt) siRNA, which is reduced to 15% in the presence of the TILRR siRNA (B), while levels of spectrin are unchanged by any of the conditions (C). B, $p < 0.05$, nt siRNA at 0 vs 30 and 60 min; nt siRNA vs TILRR siRNA at 60 min.
