# Supplementary material for: Computational Modelling of NF-κB Activation by IL-1RI and Its Co-Receptor TILRR, Predicts a Role for Cytoskeletal Sequestration of IκBα in Inflammatory Signalling
Source: PLoS One. 2015 Jun 25;10(6):e0129888. doi: 10.1371/journal.pone.0129888 (PMC4482363; doi:10.1371/journal.pone.0129888)
Supplement: S4 Fig — In vitro data obtained by single cell readings from live cells are compared with in silico simulations. (PDF) [file pone.0129888.s004.pdf]

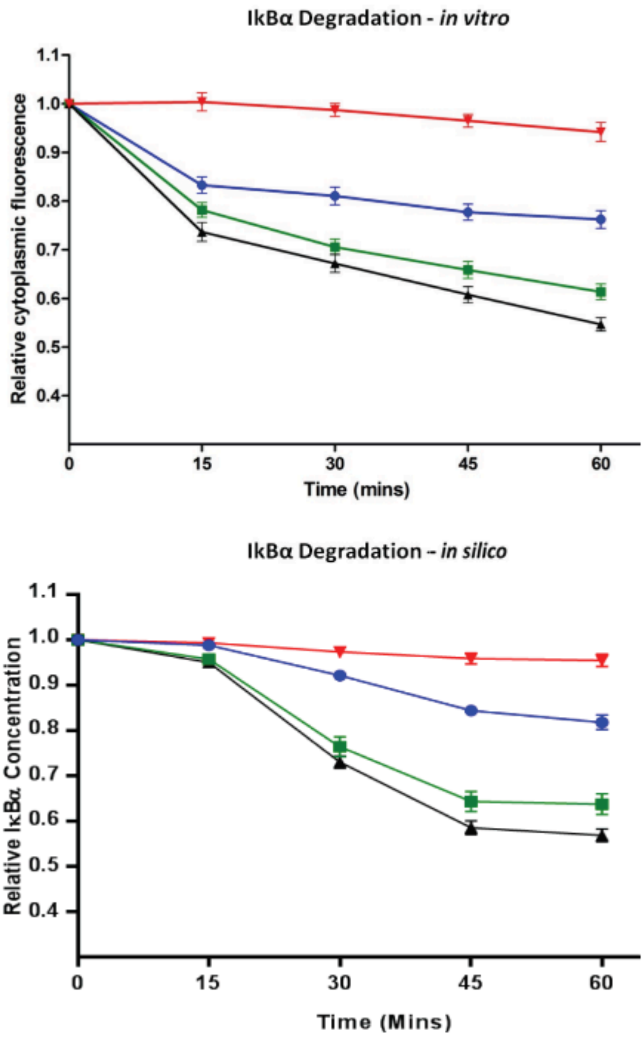


**S4 Fig. *In silico* simulations accurately reproduce data from *in vitro* experiments, demonstrating varying degrees of IκBα degradation induced by TILRR mutants.**

Top graph: *In vitro* experiments, showing IL-1-induced degradation of IκBα-EGFP in real time, as previously (24), in the presence of wild type TILRR or TILRR mutants or TILRR siRNA, as indicated. Data are expressed relative to levels at t=0; 30 cells/experiment, n=3 for each condition.

Bottom graph: *In silico* simulations of IκBα degradation under the same conditions reproduce the *in vitro* data. Results predict a pronounced reduction in IκBα degradation in the presence of the D448 mutant, corresponding to about 55% of levels recorded in the presence of wild type TILRR at 60 minutes. In comparison, substitution of residue R425 has a reduced impact on inhibitor degradation, corresponding to approximately 15%. Mean±SEM, n=4. $p < 0.0005$, WT TILRR vs TILRR D448 and TILRR siRNA at 60 min; $p < 0.05$, WT TILRR vs TILRR R425 at 60 min.

WT TILRR(▲), TILRR mut R425 (■), TILRR mut D448 (●), TILRR siRNA (▼).
