# Supplementary material for: Computational Modelling of NF-κB Activation by IL-1RI and Its Co-Receptor TILRR, Predicts a Role for Cytoskeletal Sequestration of IκBα in Inflammatory Signalling
Source: PLoS One. 2015 Jun 25;10(6):e0129888. doi: 10.1371/journal.pone.0129888 (PMC4482363; doi:10.1371/journal.pone.0129888)
Supplement: S5 Fig — Simulations comparing effects of cytoskeletal binding in the presence of wild type and mutant TILRR show no effect under any of the conditions. (PDF) [file pone.0129888.s005.pdf]

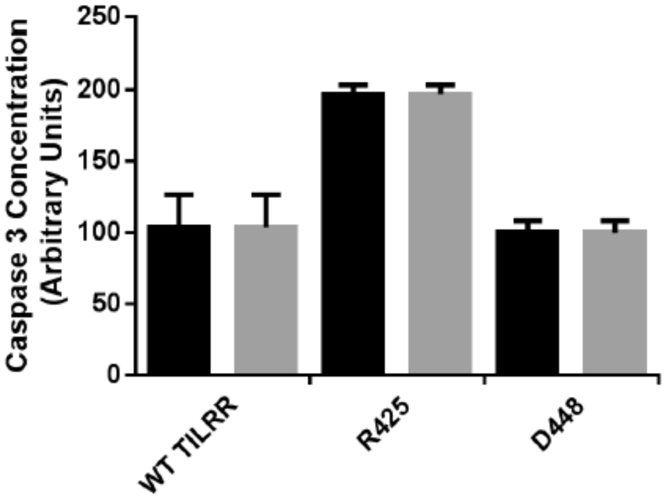

S5 Fig. Cytoskeletal sequestration and release of IκBα does not affect inhibitor degradation-independent activation of anti-apoptotic responses.

Reductions in Caspase 3 activity and cell apoptosis correspond to approximately 50% in the presence of wild type TILRR and the D448 TILRR mutant, but are unaffected by the R425 mutant (14). *In silico* simulations agree with the *in vitro* data, and show in addition, the same level of Caspase 3 activity in the presence (■) and absence (■) of cytoskeletal sequestration and release of the inhibitor, for all conditions. Mean±SEM, n=3.
