# Supplementary material for: Computational Modelling of NF-κB Activation by IL-1RI and Its Co-Receptor TILRR, Predicts a Role for Cytoskeletal Sequestration of IκBα in Inflammatory Signalling
Source: PLoS One. 2015 Jun 25;10(6):e0129888. doi: 10.1371/journal.pone.0129888 (PMC4482363; doi:10.1371/journal.pone.0129888)
Supplement: S1 Table — The Protein agent uses eight memory variables to record agent type, location and state. (PDF) [file pone.0129888.s006.pdf]

S1 Table. Protein agent memory variables

Name	Type	Description
ID	int	Agent ID
Type	int	Protein type (e.g. MyD88, inactive IKK, active IKK, etc.)
Loc	int	Cytoplasm or Nucleus (used for tracking agents)
Tag	int	Identifier for transfected proteins (for tracking)
Timer	int	Counter for timing temporary state changes
X	double	Position x axis
Y	double	Position y axis
Z	double	Position z axis
