# Supplementary material for: Computational Modelling of NF-κB Activation by IL-1RI and Its Co-Receptor TILRR, Predicts a Role for Cytoskeletal Sequestration of IκBα in Inflammatory Signalling
Source: PLoS One. 2015 Jun 25;10(6):e0129888. doi: 10.1371/journal.pone.0129888 (PMC4482363; doi:10.1371/journal.pone.0129888)
Supplement: S2 Table — Agent communications in FLAME are messages sent and received by agents. Messages from Protein agents signal their location and state, as well as requests for interactions. (PDF) [file pone.0129888.s007.pdf]

S2 Table. Protein agent messages

**Name :**		**proteinLocation**
**Description:**		Output agent memory for determining potential interactions
**Elements**		
**Name**	**Type**	**Description**
ID	int	Agent memory of same name
Type	int	Agent memory of same name
Tag	int	Agent memory of same name
Timer	int	Agent memory of same name
X	double	Agent memory of same name
Y	double	Agent memory of same name
Z	double	Agent memory of same name

**Name :**		**proteinReceptor**
**Description:**		Message from protein to notify receptor of an interaction with it, where the receptor then needs to update its own state
**Elements**		
**Name**	**Type**	**Description**
ID	int	ID of the target receptor agent
Type	int	Agent memory of same name
targetType	int	Type of the target receptor agent
Tag	int	Agent memory of same name
X	double	Agent memory of same name
Y	double	Agent memory of same name
Z	double	Agent memory of same name
