# Supplementary material for: Computational Modelling of NF-κB Activation by IL-1RI and Its Co-Receptor TILRR, Predicts a Role for Cytoskeletal Sequestration of IκBα in Inflammatory Signalling
Source: PLoS One. 2015 Jun 25;10(6):e0129888. doi: 10.1371/journal.pone.0129888 (PMC4482363; doi:10.1371/journal.pone.0129888)
Supplement: S3 Table — Agent functions are performed in a predefined order, and include outputting messages, reading messages and updating the internal state. (PDF) [file pone.0129888.s008.pdf]

S3 Table. Protein agent functions

**Name :**	**proteinTimer**
**Description:**	Update protein state on expired Timer
**Current State:**	**0**
**Next State:**	**1**
**Flow**	
Decrement Timer value -> If Timer == 0 update state (Type variable records state) -> Destroy agent if state change requires removal of the agent	
**Inputs**	
**Message Name**	**From Agent**
n/a	n/a
**Outputs**	
**Message Name**	**To Agent**
n/a	n/a

**Name :**	**proteinOutput**
**Description:**	Output agent memory
**Current State:**	**1**
**Next State:**	**2**
**Flow**	
Output proteinLocation message	
**Inputs**	
**Message Name**	**From Agent**
n/a	n/a
**Outputs**	
**Message Name**	**To Agent**
proteinLocation	Protein, Receptor

**Name :**	**Protein Receptor**
**Description:**	Calculate protein interactions with receptors
**Current State:**	**2**
**Next State:**	**3**
**Flow**	
Read receptorLocation messages -> If Receptor Type is compatible with Protein Type for interaction, calculate distance -> Identify closest Receptor within interaction range -> Update Protein memory to reflect result of interaction -> Output proteinReceptor message	
**Inputs**	
**Message Name**	**From Agent**
receptorLocation	Receptor
**Outputs**	
**Message Name**	**To Agent**
proteinReceptor	Receptor

**Name :**	**proteinMove**
**Description:**	Calculate Protein Protein interactions, update Protein location
**Current State:**	**3**
**Next State:**	**4**
**Flow**	
Read proteinLocation message -> If Protein Type is compatible with message Protein type for interaction, calculate distance -> Identify closest Protein within interaction range -> Update Protein memory to reflect result of interaction -> Destroy agent if interaction requires removal of the agent ->	

Update agent X,Y,Z coordinate position	
**Inputs**	
**Message Name**	**From Agent**
proteinLocation	Protein
**Outputs**	
**Message Name**	**To Agent**
n/a	n/a
