# Supplementary material for: Computational Modelling of NF-κB Activation by IL-1RI and Its Co-Receptor TILRR, Predicts a Role for Cytoskeletal Sequestration of IκBα in Inflammatory Signalling
Source: PLoS One. 2015 Jun 25;10(6):e0129888. doi: 10.1371/journal.pone.0129888 (PMC4482363; doi:10.1371/journal.pone.0129888)
Supplement: S1 Text — FLAME is an agent-based modelling framework designed for high performance and parallel processing of agents. (PDF) [file pone.0129888.s009.pdf]

S1 Text. The Agent-Based Modelling Framework: FLAME

The principle of agent-based modelling is that each interacting component in a complex system is represented as an autonomous ‘agent’, a software artifact that is programmed to behave exactly as the actual component does in terms of reacting to and interacting with its environment under all feasible circumstances. A number of software environments exist for agent-based modelling, but the scale of many of these models, including the one described here, requires high performance computers. FLAME is a flexible robust agent-based supercomputing modelling framework, which enables the simulation target systems (http://www.flame.ac.uk). In FLAME each agent is described in terms of a set of internal states, together with a description of its ‘internal’ memory containing information about its precise location and direction of movement etc. The model defines the type of messages that agents can send to convey information about their position, state etc. to other agents in the system. The agents will then behave according to a set of rules – functions – that determine the response under the current conditions – location, state, messages received etc. During each iteration, the simulation “visits” every agent in a random order and updates the complete system. The total behaviour of the system then emerges as a result of myriad interactions of the agents. The code generated can run on high performance computers (HPCs) and GPUs. Development of the FLAME model used GPUs and a HPC, *Iceberg*. As a node of the White Rose Computing Grid, the *Iceberg* is the Linux-based high performance computing cluster at Sheffield University, which provides ‘cloud computing’ with its INTEL-based cluster (912 INTEL cores, 8 Nvidia Tesla Fermi M2070 GPU units for GPU programming and 1920 GB memory) and AMD-based cluster (632 AMD cores and 2528 GB memory).
