# Supplementary material for: Computational Modelling of NF-κB Activation by IL-1RI and Its Co-Receptor TILRR, Predicts a Role for Cytoskeletal Sequestration of IκBα in Inflammatory Signalling
Source: PLoS One. 2015 Jun 25;10(6):e0129888. doi: 10.1371/journal.pone.0129888 (PMC4482363; doi:10.1371/journal.pone.0129888)
Supplement: S2 Text — The FLAME agent based model is developed in three steps—agent’s memory and functions, implementation of functions, and simulation of the starting state. Receptors and signalling intermediates are represented as agents and simulations describe key signalling events. (PDF) [file pone.0129888.s010.pdf]

## S2 Text. Agent Based Modelling

Agent based models are constructed from three main parts - a description of the agent types including their memory and functions, the implementation of the agent functions, which determines the rule-set for their behaviour, and for each simulation a starting state including a list of all agents. Execution of the model follows an iterative procedure, where each iteration represents a fixed time step and system update, described in Text S1.

In this agent-based model, both receptors and signalling intermediates are represented as agents and the simulation as a whole describes key signalling events occurring in a single cell. The cell is simulated as two concentric spheres, the outer representing the cell wall and the inner representing the nuclear membrane with the nucleus taking up 5% of the total cell volume (28, 29). Receptors are generally confined to a membrane such as the cell wall for the IL-1RI or the nuclear membrane for transport receptors. Proteins can move throughout the cytoplasm or the nucleus, but cannot travel through membranes without the use of a transport receptor; this prevents proteins from escaping the cell and enables selection of proteins, which are allowed to move between compartments.

Most interactions between agents involve activation. For example, an agent representing an IRAK (IL-1 receptor associated kinase) can be activated by an active MyD88-adapter protein agent, if they come in close proximity. Such chains of activation events form the core of the model pathway. For detailed descriptions, see Tables S1-S3.
