# Supplementary material for: Computational Modelling of NF-κB Activation by IL-1RI and Its Co-Receptor TILRR, Predicts a Role for Cytoskeletal Sequestration of IκBα in Inflammatory Signalling
Source: PLoS One. 2015 Jun 25;10(6):e0129888. doi: 10.1371/journal.pone.0129888 (PMC4482363; doi:10.1371/journal.pone.0129888)
Supplement: S3 Text — Free roaming proteins involved in the signal pathway act according to defined rules for Protein functions, determined by the type and state of the Protein agent. (PDF) [file pone.0129888.s011.pdf]

S3 Text. Agent Name: protein

This agent represents any of the free roaming proteins involved in the signal pathway, such as MyD88, IRAK, Ras etc. Most Protein agents are confined to the cytoplasm, however others such as IκBαand NF-κB can move to and from the nucleus via interaction with nuclear transport receptors. Protein agents can interact with both Receptor agents and other Protein agents, the rules governing which interactions are permissible are defined in the Protein functions and are determined by the type and state of the Protein agent. For example a Protein agent representing inactive IKK can only interact with Protein agents representing active forms of upstream regulators (Ras, TAK and Akt) and will change state to active IKK as a result. Active IKK can only interact with an agent representing a NF-κB: IκBα dimer and will cause the dimer to split into two separate agents representing pIκBα and NF-κB.

Protein agents can also change state based on an internal timer, which is used for a variety of processes controlling the pathway. Such timed processes include transcription, in which a new Protein agent is created at the point of transcription, but is in a state of being transcribed and unable to interact with other agents until after a set time when it changes its state to that of the complete protein.

Two agent memory variables are used purely for tracking agents during simulations, to allow for easier data acquisition. One variable, Loc, identifies the localization of each protein (nuclear or cytoplasmic) to allow easy tracking of where agents are without having to compute their coordinates. The other variable named Tag can be used to monitor levels of simulated transfections, such as transfected IκBα agents, which are identical to endogenous IκBα except for the Tag. Endogenous IκBα agents will not contain this Tag and hence the number of Tags can be used to monitor the transfected levels without compromising the function of the simulated cell.
