# Supplementary material for: Computational Modelling of NF-κB Activation by IL-1RI and Its Co-Receptor TILRR, Predicts a Role for Cytoskeletal Sequestration of IκBα in Inflammatory Signalling
Source: PLoS One. 2015 Jun 25;10(6):e0129888. doi: 10.1371/journal.pone.0129888 (PMC4482363; doi:10.1371/journal.pone.0129888)
Supplement: S4 Text — The initial state of the agents is set to an inactive form followed by two hours simulations of the un-stimulated steady state, prior to simulating activation through the IL-1 type I receptor. (PDF) [file pone.0129888.s012.pdf]

S4 Text. Steady State

In this model several types of agents can be in one of multiple states and locations even in an unstimulated cell. For example IκBα can be free, bound to the cytoskeleton, bound to NF-κB, be phosphorylated prior to degradation, or in the process of being transcribed and located in either the nucleus or cytoplasm. The parameters of the model have been trained to allow these agents to distribute into different forms, to reflect the biological data describing the system steady state in the un-stimulated cell.

In each simulation, the initial states of the agents are set to an inactive form and located in the cytoplasm. An initial simulation is run for two hours to allow the cell to reach the unstimulated steady state before a second simulation is run which introduces activation of the receptor complex.

The process has three main benefits. The initial state of the model is easily configured, as relatively few states describing agent characteristics need to be included. Further, cell behaviour can be confirmed prior to stimulation, and changes induced during stimulation are limited to activated agents and cells. Finally, maintaining a representation of unstimulated cells allows continued testing of both the activated signalling pathway and the system steady state.
