# Supplementary material for: Computational Modelling of NF-κB Activation by IL-1RI and Its Co-Receptor TILRR, Predicts a Role for Cytoskeletal Sequestration of IκBα in Inflammatory Signalling
Source: PLoS One. 2015 Jun 25;10(6):e0129888. doi: 10.1371/journal.pone.0129888 (PMC4482363; doi:10.1371/journal.pone.0129888)
Supplement: S5 Text — Reaction rates are determined by the concentration of reagents and their affinity. (PDF) [file pone.0129888.s013.pdf]

S5 Text. Agent Numbers and Affinities

Reaction rates in the agent based model are determined by two factors, the concentration of the reagents and their affinity for the reaction (the reaction rate is constant). Concentration is modelled by agent number and affinity by the distance required for agent interaction (11). Increasing the number of agents or the distance at which they are able to interact increases the reaction rate. Reactions are not stochastic and will occur between two agents capable of interacting if they are within the distance required for a given iteration.

For the majority of proteins and reactions in the model, the endogenous expression levels and reaction rates are currently unknown. Endogenous levels of NF-κB, IκBα and IL1-R1 were based on earlier estimates and agent levels were populated in appropriate proportions in the simulation (24, 28-30, Qwarnstrom EE, Page RC, Gillis S, Dower SK. Binding, internalization, and intracellular localization of interleukin-1 beta in human diploid fibroblasts. J. Biol. Chem. 1988;263(17):8261-8269).

Agents representing proteins in the signal cascade were tested over a range of concentrations and modified based on *in vitro* data. For example as the loss of Akt regulated responses in the presence of the R425 TILRR-mutant had little impact on the inflammatory response the affinity for the Akt:IKK interaction was reduced to produce the appropriate decrease, while keeping Akt concentrations high enough to control Caspase 3, using the standard reaction affinity.

Nuclear import and export receptor levels were adjusted so that free NF-κB would accumulate in the nucleus at concentrations that mimic the *in vitro* data. The affinity of nuclear promoter agents interacting with NF-κB was adjusted to induce IκBα transcription at a level, which would restore the un-stimulated steady state.

Cytoskeletal binding sites for IκBα were set to 30-fold the level of NF-κB binding, to reflect the greater abundance of cytoskeletal proteins. Affinity of the cytoskeletal binding (Kon) was set to maintain a ratio of 2:1 in the resting cell (11). The rate of release (Koff) was adjusted by modifying the affinity between the complexes and dissociation agents.
